# Frontotemporal dementia – a catastrophic form of dementia praecox

**DOI:** 10.1192/j.eurpsy.2023.1971

**Published:** 2023-07-19

**Authors:** A. R. Costa, S. Jesus, C. Vicente

**Affiliations:** 1 Department of psychiatry and mental health, Baixo Vouga Hospital Centre, Aveiro, Portugal

## Abstract

**Introduction:**

Frontotemporal dementia (FTD) is a devastating neurodegenerative condition with several clinical presentations for which there is currently no effective treatment. Although much less common than Alzheimer’s disease, the impact of FTD is high thanks to its relatively early onset and high heritability. This subtype of brain atrophy production decided the frontal and temporal lobes.

Clinical heterogeneity and overlap with other neurodegenerative and psychiatric syndromes complicate diagnosis. Three different subtypes are recognized: behavioral variant, non-fluent aphasia, and progressive semantic dementia.

**Objectives:**

Clinical review of frontotemporal dementia including the clinics, determination of diagnosis, treatment, and prognosis with a clinical case report.

**Methods:**

Bibliographic research with the terms dementia, frontotemporal dementia.

**Results:**

The current clinical case follows a patient in her fifties, born in Brazil, who has a child and a poor social support network. No significant history, celebrating at least two years marked by an evolution framework of progressive change in verbal memory, increase in verbal influence, change in executive functions, namely, and definition of verbal decision.

**Conclusions:**

In general terms, behavioral and language alterations are the dominant aspects of this type of dementia and as characteristics common to the various subgroups of FTD.

FTD is a catastrophic clinical entity thanks to its beginning, the exuberance of the clinical picture, and mainly the lack of treatment with guidance aimed at relieving symptoms and improving the patient’s quality of life.

**Disclosure of Interest:**

None Declared

